# OLDER ADULTS INFLUENCING THE CIVIC ENGAGEMENT OF VOLUNTEER CAREGIVERS DURING THE COVID-19 PANDEMIC

**DOI:** 10.1093/geroni/igac059.2945

**Published:** 2022-12-20

**Authors:** Kathleen Gale

**Affiliations:** Eras Senior Network Inc., Waukesha, Wisconsin, United States

## Abstract

Older adults who do not qualify for government entitlement programs but lack sufficient financial or other personal resources help them to age in place rely on community organizations to assist them with social determinants of health. However, these organizations struggle with high rates of volunteer caregiver turnover, a looming crisis spotlighted during the COVID-19 pandemic. At least one group of volunteer caregivers continued to serve during this period. This phenomenological study investigated the experiences of eight volunteer caregivers who served older adults through a Faith in Action model volunteer driver program during the first year of the pandemic. Findings indicate that a volunteer’s commitment to older adults, awareness of the needs of older adults, and established relationships with older adults were more important than concerns about COVID-19. Study participants were alert to the needs of older adults during and between service activities, making personal sacrifices of time, money, and physical exertion to accommodate needs. Participants approached service caring for the older adult and expected reciprocal care in the form of appreciation and respect. They considered discontinuing service if efforts weren’t appreciated or if deeply held values were violated. The volunteer service organization was key in mitigating conflict within the relationship of the volunteer and older adult, thereby increasing the likelihood that the volunteer would continue serving. Implications include training older adult clients to meet expectations of care and appreciation for volunteers, managing volunteers whose personal sacrifices exceed the scope of volunteer service, and supporting volunteers whose values have been violated.

